# Supplementary material for: The Pseudomonas aeruginosa membrane histidine kinase BqsS/CarS directly senses environmental ferrous iron (Fe2+)
Source: J Biol Chem. 2025 Nov 5;301(12):110801. doi: 10.1016/j.jbc.2025.110801 (PMC12666569; doi:10.1016/j.jbc.2025.110801)
Supplement: Supporting information [file mmc1.docx]

**SUPPORTING INFORMATION**

The *Pseudomonas aeruginosa* Membrane Histidine Kinase BqsS/CarS Directly Senses Environmental Ferrous Iron (Fe^2+^)

Alexander Paredes^1^, Chioma Iheacho^1^, Kelly N. Chacón^2^, and Aaron T. Smith^1*^

^1^Department of Chemistry and Biochemistry, University of Maryland, Baltimore County, Baltimore, Maryland, 21250 USA

^2^Department of Chemistry, Reed College, Portland, Oregon, 97202 USA

^*^To whom correspondence should be addressed. Tel: 410-455-1985; E-mail: smitha@umbc.edu

**Figure S1**. Detergent solubilization tests of P. aeruginosa BqsS. Normalized western blot analysis of the efficacy of various detergents to solubilize intact PaBqsS (molecular weight of ca. 52 kDa). Of the detergents tested, LDAO, DDM, and Fos-Choline-14 showed the highest solubilization efficacy compared to the SDS control (far right lane). Ultimately, Fos-Choline-14 was chosen due to its ability to solubilize PaBqsS stably and homogenously.


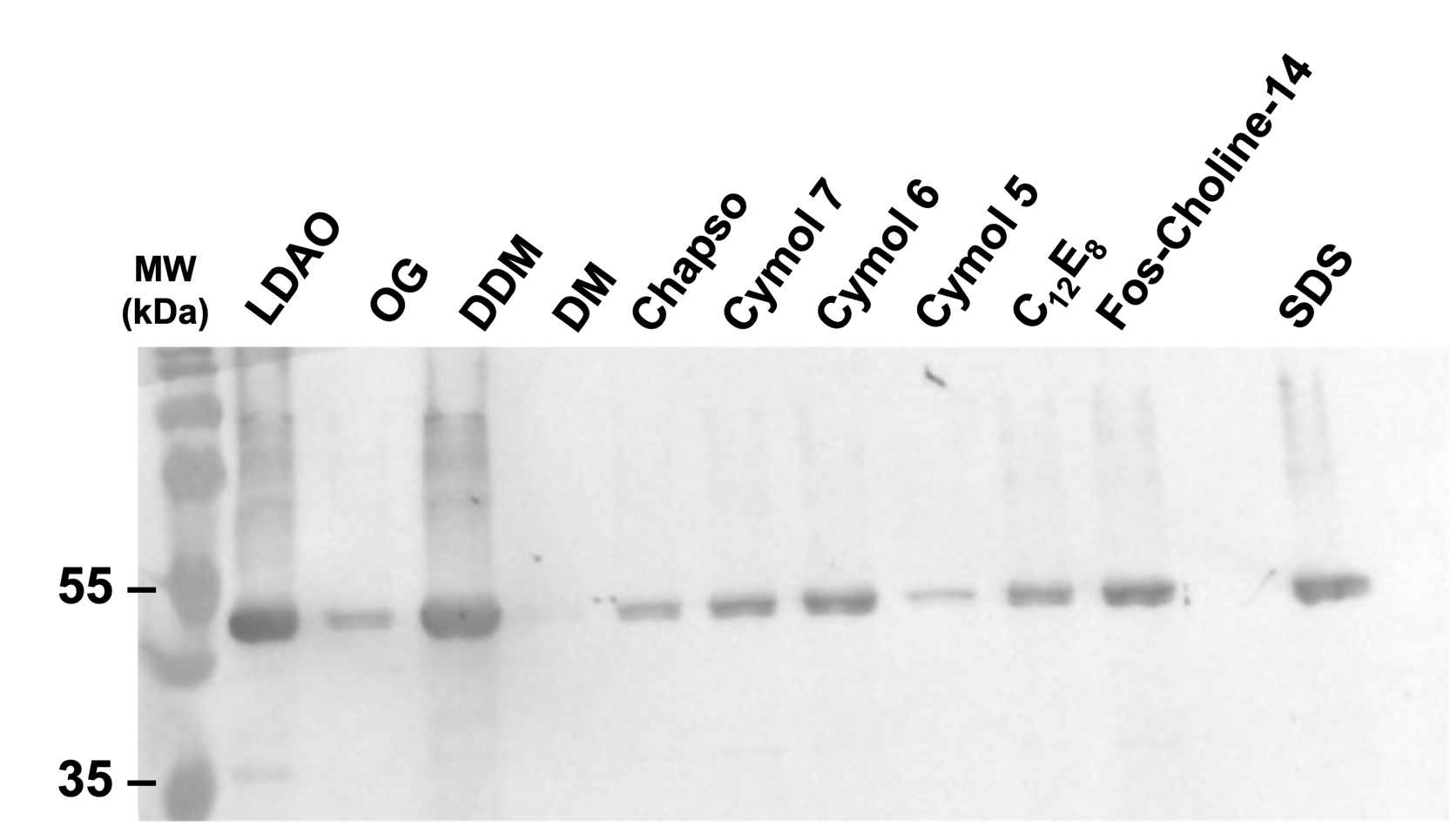

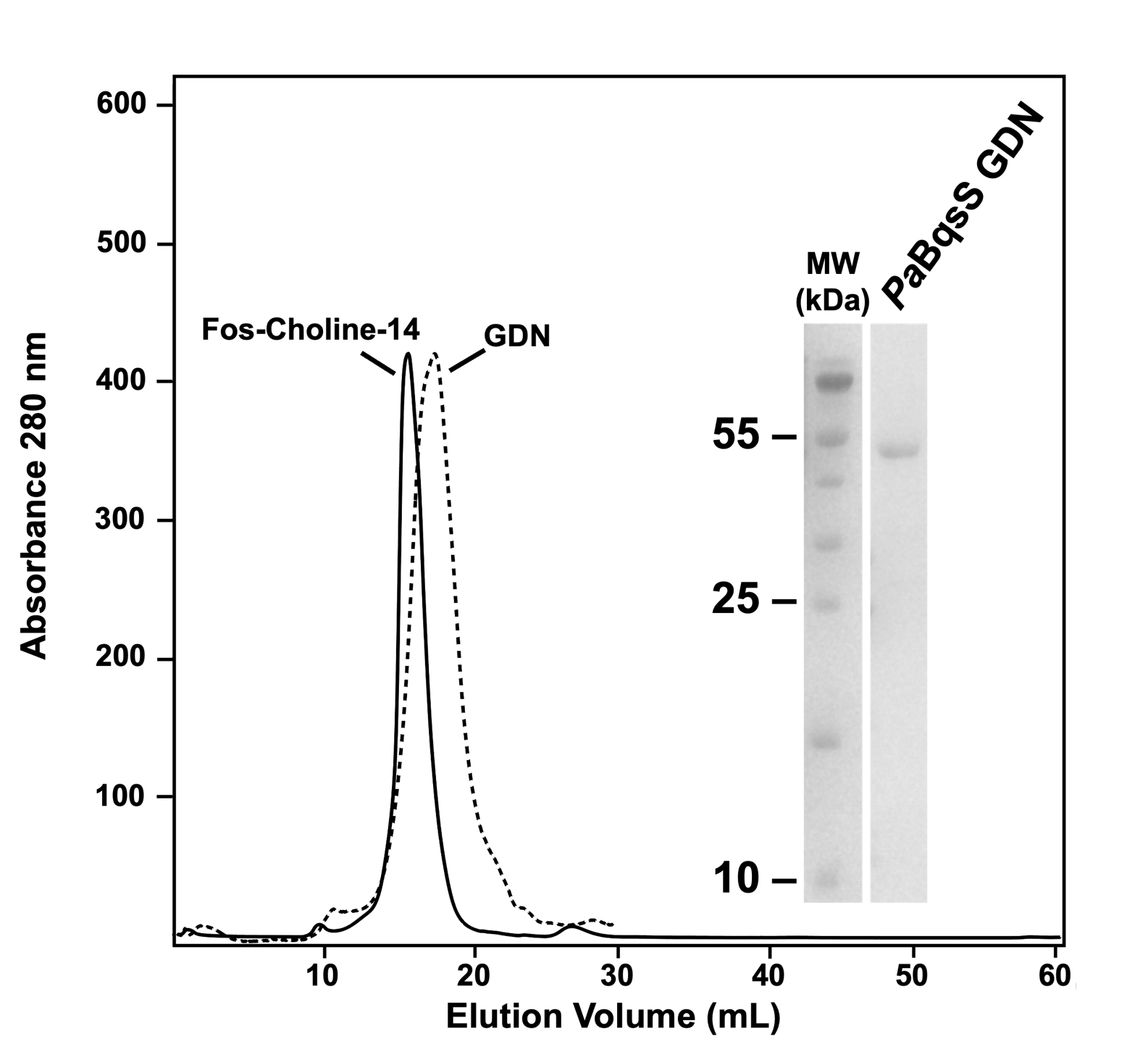


**Figure S2**. Size-exclusion chromatography is most consistent with purified PaBqsS solubilized in Fos-Choline-14 existing as a dimer of dimers (solid), while PaBqsS solubilized in glyco-diosgenin (GDN) is most consistent with a dimeric quaternary structure (dashed). Inset. 15 % SDS-PAGE analysis of PaBqsS after solubilization and purification in GDN.

**Figure S3**. Mass profile of WT PaBqsS in Fos-Choline-14 determined by mass photometry. Empty Fos-Choline-14 micelles are observed at ca. 53 kDa, while dimeric WT PaBqsS is observed at ca. 96 kDa. The molecular weights of the gaussian fits were determined by use of a calibration curve of two standards: β-amylase and thyroglobulin.


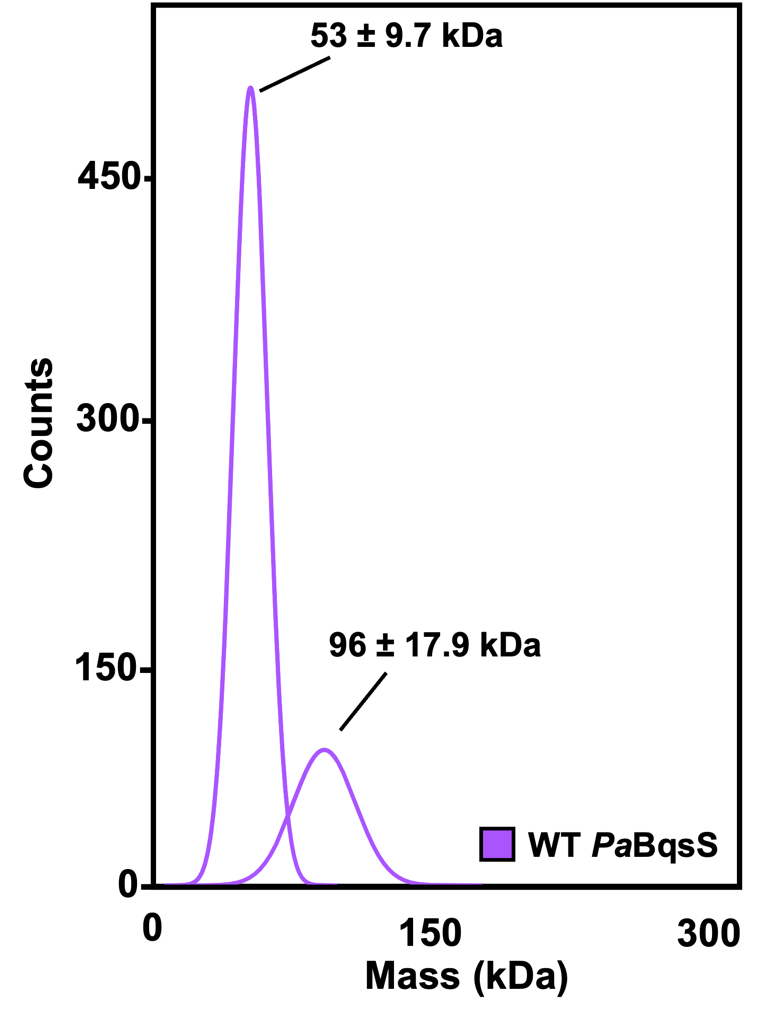


**Figure S4**. AlphaFold model of intact, dimeric PaBqsS color-coded with the per-residue confidence score (plDDT) from very low confidence (orange) to very high confidence (blue).

**Figure S5**. WT PaBqsS and PaBqsS variants E45A, E48A, and N49A have similar purities in Fos-Choline-14 (inset; 15% SDS-PAGE), and all migrate identically based on size-exclusion chromatography.

**Figure S6**. The pre-edge features found in the normalized X-ray absorption near-edge structure (XANES) spectrum of intact, Fe^2+^-bound WT PaBqsS (purple), E45A PaBqsS (orange), and N49A PaBqsS (green). Inset: full XANES spectra of intact, Fe^2+^-bound WT PaBqsS (purple), E45A PaBqsS (orange), and N49A PaBqsS (green).


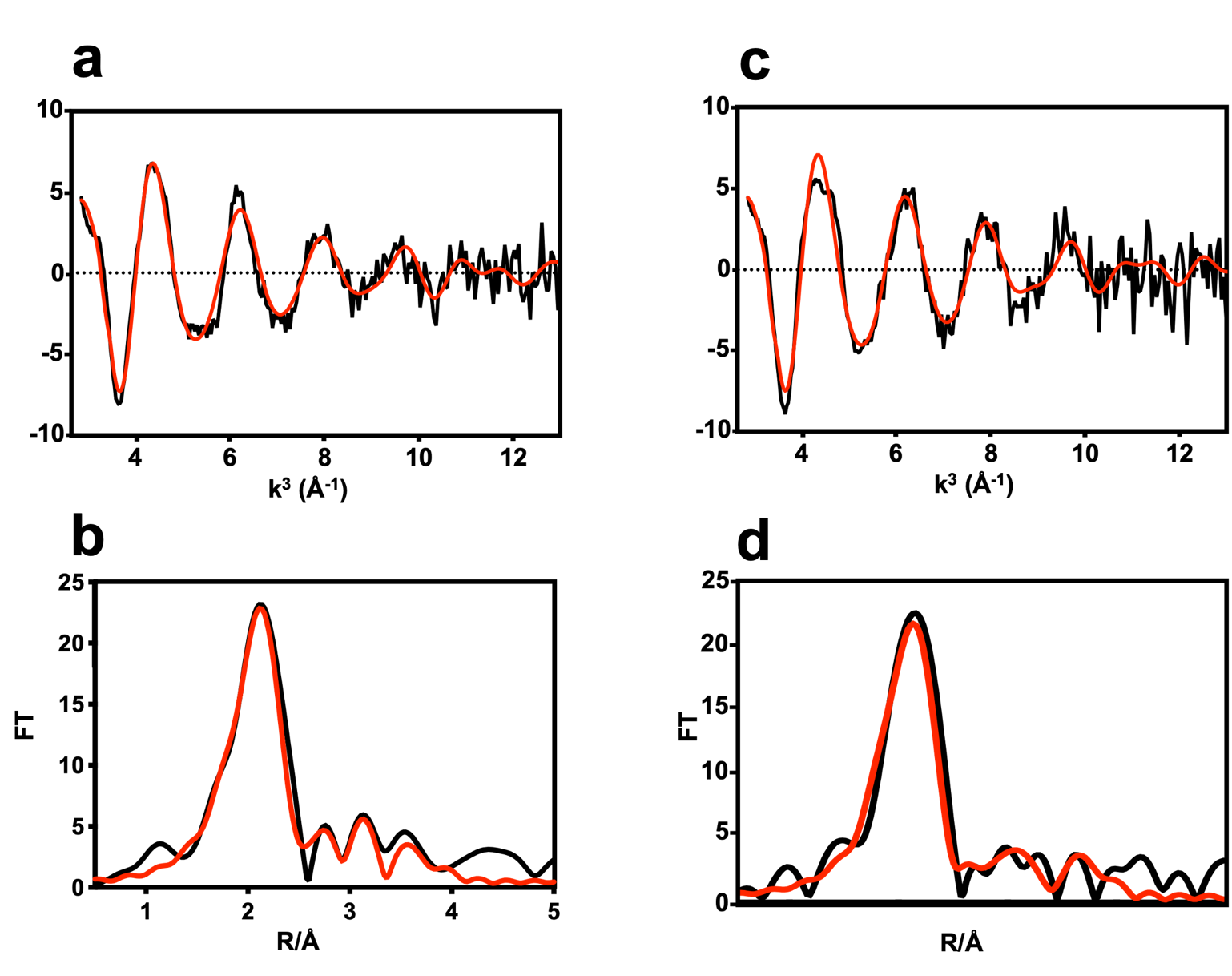


**Figure S7**. The processed WT PaBqsS EXAFS (**a**) and resulting Fourier transformed data (**b**) are consistent with the presence of Fe^2+^ in an octahedral geometry composed of an N/O-rich environment. The processed N49A PaBqsS EXAFS (**c**) and resulting Fourier transformed data (**d**) suggest a nearly identical ligation sphere. The black traces represent the experimental data while the red traces represent the EXCURVE-fitted data.

**Figure S8**. Modeling and purification of the cytosolic DHp and CA domains of PaBqsS (cytoBqsS). **a**. The AlphaFold model of intact PaBqsS with the various domains (periplasmic, DHp, CA) color-coded. Inset. The cytoplasmic domain of PaBqsS (cytoBqsS) consists of a dimerization and His phosphotransfer (DHp) domain (pink) that houses a conserved phosphate-accepting His residue (His^239^) and a catalytic ATP-binding domain (CA) domain (orange) that catalyzes the transfer of the γ-phosphate from ATP to His^239^. **b**. Size-exclusion chromatogram (SEC) and 15 % SDS-PAGE analysis (inset) of cytoBqsS (molecular weight of ca. 27 kDa).
