# Supplementary material for: The Pseudomonas aeruginosa membrane histidine kinase BqsS/CarS directly senses environmental ferrous iron (Fe2+)
Source: J Biol Chem. 2025 Nov 5;301(12):110801. doi: 10.1016/j.jbc.2025.110801 (PMC12666569; doi:10.1016/j.jbc.2025.110801)
Supplement: Table S1 [file mmc2.docx]

|  |  | **Fe-O/N (non His)** | | | **Fe-C (non His)** | | | **E_o_^e^ (eV)** |
| --- | --- | --- | --- | --- | --- | --- | --- | --- |
| **Sample** | **Fit index^a^** | **No^b^** | **R^c^ (Å)** | **DW^d^ (Å^2^)** | **No** | **R (Å)** | **DW (Å^2^)** |  |
| WT *Pa*BqsS + Fe^2+^ | 0.076 | 2.0 | 2.14 | 0.008 | 2.0 | 2.89 | 0.013 | -4.77 |
|  |  | 4.0 | 2.04 | 0.022 | 2.0 | 3.09 | 0.004 |  |
|  |  |  |  |  | 2.0 | 3.70 | 0.004 |  |
|  |  |  |  |  |  |  |  |  |
| N49A *Pa*BqsS + Fe^2+^ | 0.10 | 2.0 | 2.17 | 0.005 | 2 | 2.96 | 0.027 | -3.23 |
|  |  | 4.0 | 2.06 | 0.016 | 2 | 3.10 | 0.001 |  |
|  |  |  |  |  | 2 | 3.76 | 0.003 |  |
| ^a^The least-squares fitting parameter (see *Methods*) ^b^Coordination number ^c^Bond length ^d^Debye-Waller factor ^e^Photoelectron energy threshold | | | | | | | | |

**Table S1.** Fits obtained for the Fe K-EXAFS of WT PaBqsS and N49A PaBqsS bound to Fe^2+^. Fitted EXAFS data suggest a nearly identical ligation sphere between WT PaBqsS and N49A PaBqsS. Fits were determined by curve fitting using the program EXCURVE (version 9.2).
