# Supplementary material for: The Pseudomonas aeruginosa membrane histidine kinase BqsS/CarS directly senses environmental ferrous iron (Fe2+)
Source: J Biol Chem. 2025 Nov 5;301(12):110801. doi: 10.1016/j.jbc.2025.110801 (PMC12666569; doi:10.1016/j.jbc.2025.110801)
Supplement: Table S2 [file mmc3.docx]

| **Name** | **Description** | **Oligonucleotide sequence** |
| --- | --- | --- |
| E45A_Fwd | Used for A^45^xxE^48^ site-directed mutagenesis | 5’ -GTTCTCCGCTTCCGCACGCAGGTTGCC -3’ |
| E45A_Rev | Used for A^45^xxE^48^ site-directed mutagenesis | 5’-GGCAACCTGCGTGCGGAAGCGGAGAAC-3’ |
| E48A_Fwd | Used for E^45^xxA^48^ site-directed mutagenesis | 5’-CAACCAGCAGGTTCGCCGCTTCCTCACGC-3’ |
| E48A_Rev | Used for E^45^xxA^48^ site-directed mutagenesis | 5’-GCGTGAGGAAGCGGCGAACCTGCTGGTTG-3’ |
| N49A_Fwd | Used for E^45^xxE^48^A^49^ site-directed mutagenesis | 5’- ATCGCAACCAGCAGGGCCTCCGCTTCCTCACG -3’ |
| N49A_Rev | Used for E^45^xxE^48^A^49^ site-directed mutagenesis | 5’- CGTGAGGAAGCGGAGGCCCTGCTGGTTGCGAT -3’ |

**Table S2**. List of primers and DNA sequences used in this study. Underlines indicate the modified bases for the introductions of mutations.
